# Effect of transversus abdominis plane blocks in abdominoplasties on postoperative outcomes

**DOI:** 10.1093/bjsopen/zraf067

**Published:** 2025-06-20

**Authors:** Sascha Wellenbrock, Bettina Zidek, Laetitia S Chiarella, Fabian Nehls, Mirkka Hiort, Vanessa Neef, Tobias Hirsch, Matthias Maas, Maximilian Kueckelhaus

**Affiliations:** Department of Plastic Surgery, University Hospital Muenster, Muenster, Germany; Department of Plastic and Reconstructive Surgery, Institute for Musculoskeletal Medicine, University of Muenster, Muenster, Germany; Department of Plastic, Reconstructive and Aesthetic Surgery, Hand Surgery, Fachklinik Hornheide, Muenster, Germany; Department of Plastic Surgery, University Hospital Muenster, Muenster, Germany; Department of Plastic and Reconstructive Surgery, Institute for Musculoskeletal Medicine, University of Muenster, Muenster, Germany; Department of Plastic, Reconstructive and Aesthetic Surgery, Hand Surgery, Fachklinik Hornheide, Muenster, Germany; Department of Plastic Surgery, University Hospital Muenster, Muenster, Germany; Department of Plastic and Reconstructive Surgery, Institute for Musculoskeletal Medicine, University of Muenster, Muenster, Germany; Department of Plastic, Reconstructive and Aesthetic Surgery, Hand Surgery, Fachklinik Hornheide, Muenster, Germany; Department of Plastic Surgery, University Hospital Muenster, Muenster, Germany; Department of Plastic and Reconstructive Surgery, Institute for Musculoskeletal Medicine, University of Muenster, Muenster, Germany; Department of Plastic, Reconstructive and Aesthetic Surgery, Hand Surgery, Fachklinik Hornheide, Muenster, Germany; Department of Plastic Surgery, University Hospital Muenster, Muenster, Germany; Department of Plastic and Reconstructive Surgery, Institute for Musculoskeletal Medicine, University of Muenster, Muenster, Germany; Department of Plastic, Reconstructive and Aesthetic Surgery, Hand Surgery, Fachklinik Hornheide, Muenster, Germany; Department of Anaesthesiology, Intensive Care Medicine and Pain Therapy, Goethe University Frankfurt, University Hospital Frankfurt, Frankfurt, Germany; Department of Plastic Surgery, University Hospital Muenster, Muenster, Germany; Department of Plastic and Reconstructive Surgery, Institute for Musculoskeletal Medicine, University of Muenster, Muenster, Germany; Department of Plastic, Reconstructive and Aesthetic Surgery, Hand Surgery, Fachklinik Hornheide, Muenster, Germany; Department of Anaesthesiology, Intensive Care Medicine and Pain Therapy, Fachklinik Hornheide, Muenster, Germany; Department of Plastic Surgery, University Hospital Muenster, Muenster, Germany; Department of Plastic and Reconstructive Surgery, Institute for Musculoskeletal Medicine, University of Muenster, Muenster, Germany; Department of Plastic, Reconstructive and Aesthetic Surgery, Hand Surgery, Fachklinik Hornheide, Muenster, Germany

## Abstract

**Background:**

Acute postoperative pain after surgery may lead to significant complications including chronification of pain, impaired cardiopulmonary function, and increased healthcare costs. As a common and complex procedure, abdominoplasty is a key focus for pain management strategies. Although transversus abdominis plane blocks, which target the abdominal wall's sensory nerves to reduce postoperative pain by blocking nociceptive input, have shown promise in reducing postoperative pain in abdominal surgeries, their use in abdominoplasty remains underexplored.

**Methods:**

Outcomes for patients undergoing abdominoplasty between 2013 and 2024 were analysed, comparing those who received a transversus abdominis plane block with those who did not. Postoperative analgesia followed a standardized protocol using oral narcotics and piritramide. Pain outcomes were assessed in both groups via chart review. The primary outcome, length of hospital stay, was analysed by multivariable linear regression adjusted for patient and surgical factors. Secondary outcomes, including complications and revision rates, were assessed by logistic regression. Exploratory analyses examined how reductions in medication use affected length of hospital stay and discharge timing.

**Results:**

Overall, 192 patients who had an abdominoplasty were included in analyses: 93 had a transversus abdominis plane block and 99 did not. The transversus abdominis plane group had a significantly shorter hospital stay, with a reduction of 2.21 (95% confidence interval (c.i.) −3.07 to −1.36) days after adjusting for confounders (*P* < 0.001; effect size, Cohen's d 0.45). Surgical complications occurred in 46.9% of patients. The overall complication risk in the transversus abdominis plane block group was significantly reduced by 52% (adjusted odds ratio 0.44, 95% c.i. 0.23 to 0.84; *P* = 0.012; effect size 0.52), particularly the occurrence of haematoma (adjusted odds ratio 0.34; *P* = 0.031; effect size 0.66). Additionally, patients who had a transversus abdominis plane block required less postoperative medication, including lower tilidine (*P* = 0.038) and metamizole (*P* = 0.032) doses.

**Conclusion:**

Use of the transversus abdominis plane block in patients who had an abdominoplasty was associated with improved postoperative outcomes, highlighting its potential as an effective pain management strategy and supporting its broader clinical application.

## Teaser

This retrospective cohort study evaluated the impact of the transversus abdominis plane (TAP) block on postoperative outcomes in patients who had an abdominoplasty. The results showed that the TAP block significantly reduces length of hospital stay, lowers complication rates, including that of haematoma, and decreases postoperative medication use. These findings suggest that the TAP block is an effective regional anaesthesia technique for improving recovery after abdominoplasty procedures.

## Introduction

Acute pain is, by definition, ‘the normal, predicted physiological response to an adverse chemical, thermal, or mechanical stimulus associated with surgery, trauma, and acute illness’^1^. Postoperative pain, an inevitable side-effect of surgery, is an ongoing issue as it can cause impairment of cardiopulmonary function^2,3^, contribute to chronification and thus to an increase in cost of healthcare^4,5^, increase subsequent psychiatric diagnoses^6^, and lead to increased fatalities^7^. In the past two decades, efforts have been taken to evolve postoperative pain management, emphasizing multimodal concepts of opioid-sparing strategies^8,9^, perioperative non-pharmacological interventions^10^, and the implementation of tailored regional anaesthesia techniques in patients undergoing various complex surgeries^11,12^.

In addition to expanding the range of available postoperative pain therapies, several systematic reviews and guidelines have underscored the need for procedure-specific pain management, for example as seen in the PROSPECT projects^13–15^.

Abdominoplasty, commonly known as a ‘tummy tuck’, is one of the most frequently performed plastic surgery procedures worldwide, with 1 153 539 surgeries in 2023 carried out globally^16^. It involves removing excess skin and fat from the abdominal area, leading to an improved abdominal contour^17^.

Efforts to evaluate and detect inadequate postoperative pain management in abdominoplasties have been a central focus of perioperative care, as evidenced by the papers on quality improvement in postoperative pain management^18,19^. Regional anaesthesia techniques for abdominal procedures, including the transversus abdominis plane (TAP) block, have gained traction as effective methods for pain management. Although TAP blocks have been described extensively for various abdominal surgeries^20–23^, the literature on their use in abdominoplasties is scarce, primarily consisting of single-case reports or studies with small patient cohorts that highlight their benefits. Postoperative pain management is vital not only for reducing complications, morbidity, and mortality rates but also for enhancing rehabilitation capacity and addressing the economic burden associated with prolonged hospitalization. The aim of this study was to evaluate the effect of TAP blocks in abdominoplasty on key outcomes such as length of hospital stay (LOS), surgical complications, and postoperative analgesia usage.

## Methods

### Ethics

This study was approved by the local institutional review board (2024-409-f-S). The need for informed consent was waived owing to the nature of this retrospective cohort study, in accordance with institutional and national ethical standards.

### Study design and population

**Fig. 1:**
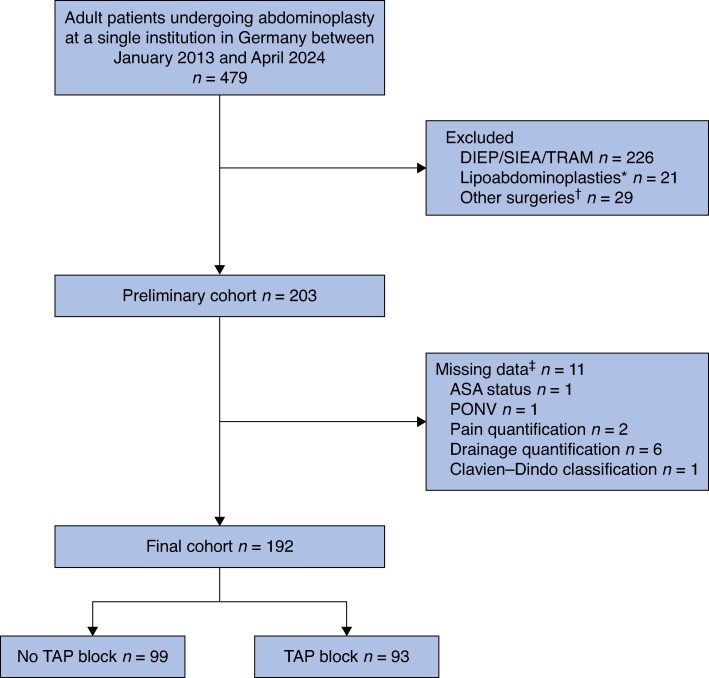
Flow chart of patient selection process and exclusion criteria *Lipoabdominoplasties > 250 ml; †other surgeries; ‡missing data; DIEP, deep inferior epigastric perforator; SIEA, superficial inferior epigastric artery; TRAM, transverse rectus abdominis myocutaneous; ASA, American Society of Anesthesiologists; PONV, postoperative nausea and vomiting; TAP, transversus abdominis plane.

All patients who had abdominoplasties at a single institution between January 2013 and April 2024 were identified. Inclusion criteria were age ≥ 18 years and an American Society of Anesthesiologists (ASA) grade of I–III. Exclusion criteria were: having previously undergone abdominoplasty as part of an autologous breast reconstruction procedure, such as deep inferior epigastric perforator flap, superficial inferior epigastric perforator flap or transverse rectus abdominis myocutaneous flap; lipoabdominoplasty involving a volume greater than 250 ml; or the abdominoplasty being performed in conjunction with abdominal hernia repair or tumour resection. Patients with missing data regarding LOS, intraoperative and postoperative medication use, and surgical complications were also excluded (*Fig. 1*).

Patients were divided into two groups based on whether or not they received a TAP block before surgery.

Postoperative pain medication and mobilization were administered according to the institution’s standard recovery protocol.

The data set was derived from the hospital’s medical records and included deidentified patient-, procedure-, and outcome-related data.

### Outcome measures

The primary outcome was LOS, defined as the number of days from the date of surgery to the date of discharge. Secondary outcomes were postoperative analgesia usage and the incidence of surgical complications. Surgical complications were defined as any adverse event requiring medical or surgical intervention; they were categorized into minor or major complications based on the Clavien–Dindo classification, and included wound healing disorders, seroma, haematoma, infection, fat necrosis, postoperative bleeding, thrombosis, abscess, and fistula. Minor complications were those classified as Clavien–Dindo grades I and II, indicating no requirement for additional surgical intervention.

### TAP block technique and procedure

All patients had a standardized protocol of general anaesthesia with endotracheal intubation. Induction was administered with intravenous sufentanil (0.3–0.4 μg/kg) and propofol (23 mg/kg), and rocuronium (0.6 mg/kg) was administered to facilitate intubation. For prevention of postoperative vomiting, all patients received 4 mg dexamethasone before surgery.

Maintenance of anaesthesia was achieved with propofol (5–10 mg per kg per hour (h)) or sevoflurane (0.7–1.0 minimal alveolar concentration). Throughout the procedure, standard monitoring was carried out, including electrocardiography, non-invasive blood pressure measurement, and pulse oximetry, and end-tidal carbon dioxide monitoring.

**Fig. 2:**
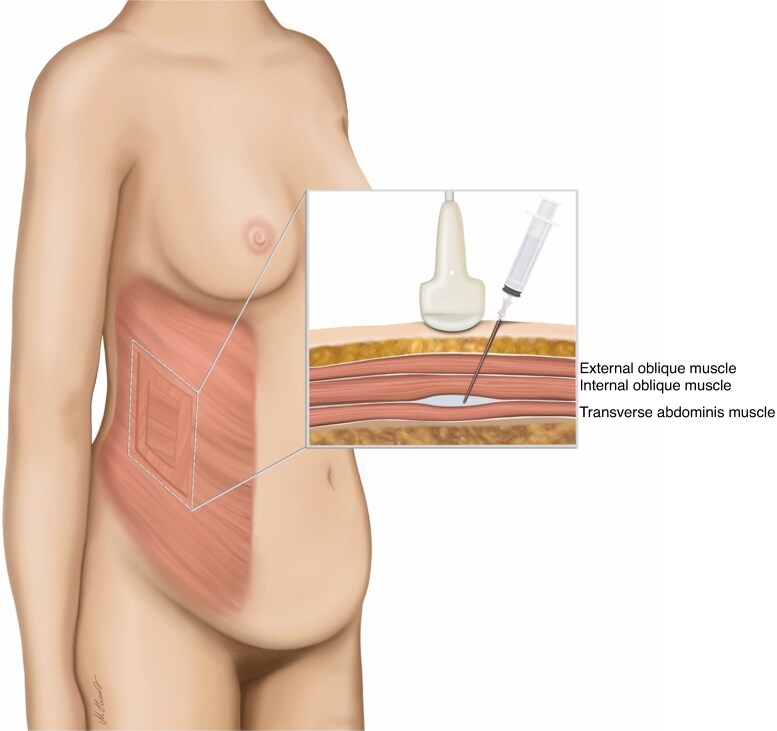
Illustration of injection technique and anatomical landmarks

When a TAP block was used, it was performed after induction of anaesthesia and before proceeding to the operating theatre. Adhering to the protocol of Hebbard *et al*.^24^, initially an ultrasound transducer was positioned transversely on the anterior axillary line as an anatomical landmark, between the costal margin and the anterior superior iliac spine. Real-time ultrasound imaging was used to visualize the muscles of the anterior abdominal wall, including the external oblique, internal oblique, and transversus abdominis. Following aseptic preparation of the injection site, a 22-G, 80-mm needle (Ultraplex^®^ 360, 30°, 22 G × 3 1/8″, 0.7 × 80 mm; B. Braun, Melsungen, Germany) was inserted medially, using an in-plane ultrasound technique to maintain continious visualization of its trajectory, advancing until the tip was located between the internal oblique and transversus abdominis muscles. After confirming correct needle placement with negative aspiration and instillation of a few millilitres of 0.9% sodium chloride, 20 ml 0.375% ropivacaine with adrenaline (5 µg/ml) was injected in 5-ml increments. When correctly positioned, the injectate was seen spreading in the transversus abdominis plane as an oval shape. The distribution of local anaesthetic between the internal oblique and transversus abdominis muscles was observed in real time to prevent an intramuscular injection. The procedure was performed bilaterally, with a total of 40 ml 0.375% ropivacaine with adrenaline (5 µg/ml) administered. *Figure 2* illustrates the injection technique and the corresponding anatomical landmarks.

### Statistical analysis

Included patients were divided into two groups: those in the TAP group received a TAP block and those in the non-TAP group did not. For the primary analysis, LOS was treated as a continuous variable. The association between perioperative variables and LOS was evaluated using multivariable linear regression. Models were adjusted for key confounders, including age, sex, body mass index (BMI), ASA grade, and the type of abdominoplasty performed, categorized as horizontal, inverted-T, panniculectomy, miniabdominoplasties, Lockwood method, or other variations.

Secondary analyses focused on the incidence of surgical complications Potential associations between complications and the use of the TAP block were evaluated using multivariate logistic regression to compare the TAP and non-TAP groups.

*Post hoc* exploratory analyses were undertaken to examine the relationships between key outcomes, including the most common complications contributing to the composite secondary outcome, the rate of acute revisional surgery, and reductions in intraoperative and postoperative medication use. These analyses were conducted using multivariate logistic and linear regression models, adjusted for the same confounder model as in the primary analysis.

All statistical analyses were carried out using Stata^®^ version 16 (StataCorp, College Station, TX, USA) and R version 4.4.2 (R Foundation for Statistical Computing, Vienna, Austria). The results of the logistic models are presented as odds ratios (ORs) or regression coefficients with corresponding 95% confidence intervals and *P* values where applicable. A significance threshold of *P* < 0.050 was applied for all analyses, with Bonferroni correction implemented to account for multiple comparisons.

## Results

### Patient demographics

After applying inclusion and exclusion criteria, the final cohort comprised 192 patients, divided into two groups based on whether or not they received a TAP block before surgery. A graphical representation of the cohort selection process is depicted in *Fig. 1*.

The study cohort consisted of 192 patients, with 99 (51.6%) in the non-TAP group and 93 (48.4%) in the TAP group. The surgical procedures included horizontal abdominoplasties (37.0%), inverted-T abdominoplasties (46.9%), panniculectomies (13.0%), and other types, such as miniabdominoplasties and Lockwood abdominoplasties (3.1%). The mean(standard deviation, s.d.) age of all patients was 40.85(12.46) years, the majority being women (79.2%). There was no significant difference in age between the two study groups. However, the non-TAP group had a significantly higher proportion of male patients compared with the TAP group (29 *versus* 12%; *P* = 0.003). No statistically significant differences were observed in BMI or ASA status between the two groups.

Patients in the TAP group had significantly fewer severe complications classified as Clavien–Dindo grade IIIb (2 *versus* 15%; *P* = 0.012) and were more likely to have no complications than the non-TAP group (62 *versus* 44%; *P* = 0.012). The mean(s.d.) numeric pain rating scale (NRS) score upon arrival in postoperative recovery was slightly lower in the TAP group (2.96(2.76) *versus* 3.16(2.72)), although this difference was not statistically significant. Conversely, the mean NRS at the time of discharge from recovery to the normal ward was significantly higher in the TAP group than in the non-TAP group (1.95(1.36) *versus* 1.30(1.27)). Group differences are depicted in *Table 1*.

**Table 1:** Patient characteristics, surgical outcomes, and co-morbidities in the cohort, comparing those who received a TAP block and those who did not

	Cohort (*n* = 192)	Non-TAP (*n* = 99)	TAP (*n* = 93)	P*
Age (years), mean(s.d.)	40.9 (12.5)	40.6 (12.7)	41.1 (12.3)	0.800†
**Sex**				0.003
Female	152 (79.17%)	70 (71%)	82 (88%)	
Male	40 (20.8%)	29 (29%)	11 (12%)	
BMI (kg/m^2^), mean(s.d.)	27.5 (5.0)	27.2 (5.2)	27.8 (4.8)	0.173†
**ASA grade**				0.310
I	53 (27.6%)	31 (31%)	22 (24%)	
II	126 (65.6%)	60 (61%)	66 (71%)	
III	13 (6.8%)	8 (8%)	5 (5%)	
**Clavien–Dindo classification**				0.012
0	102 (53.1%)	44 (44%)	58 (62%)	
I	50 (25.0%)	27 (27%)	21 (23%)	
II	6 (3.1%)	5 (5%)	3 (3%)	
IIIa	17 (8.9%)	8 (8%)	9 (10%)	
IIIb	17 (8.9%)	15 (15%)	2 (2%)	
**Co-morbidities**				
Hypothyroidism	56 (29.2%)	28 (28%)	28 (30%)	0.782
Diabetes	19 (9.9%)	11 (11%)	8 (9%)	0.560
Hypertension	47 (24.5%)	27 (27%)	20 (22%)	0.352
**Pulmonary diseases**				0.591
Asthma	17 (8.9%)	9 (9%)	8 (9%)	
COPD	2 (1.0%)	2 (2%)	0 (0%)	
Others	2 (1.0%)	1 (1%)	1 (1%)	
**Psychiatric diseases**				
Depression	19 (9.9%)	9 (9%)	10 (11%)	0.582
Others	1 (0.5%)	1 (1%)	0 (0%)	
**Neurological diseases**				
Disc prolapse	8 (4.2%)	5 (5%)	3 (3%)	0.334
Epilepsy	3 (1.6%)	3 (3%)	0 (0%)	
Other	7 (3.7%)	4 (4%)	3 (3%)	
Chronic pain	46 (24.0%)	24 (24%)	22 (24%)	0.923
**Smoking**	48 (25.0%)	22 (22%)	26 (28%)	0.361
No. of cigarettes/day				0.632
< 10	16 (8.3%)	6 (6%)	10 (11%)	
10–20	27 (14.1%)	13 (13%)	14 (15%)	
> 20	5 (2.6%)	3 (3%)	2 (2%)	
**Conservative adiposity treatment**				0.083
Bariatric	43 (22.4%)	16 (16%)	27 (29%)	
Nutrition	127 (66.2%)	68 (69%)	59 (63%)	
Aesthetic	2 (1.0%)	2 (2%)	0 (0%)	
After pregnancy	14 (7.3%)	8 (8%)	6 (6%)	
Others	6 (3.1%)	5 (5%)	1 (1%)	
**Surgical obesity treatment**				0.091
Bypass	40 (20.8%)	83 (84%)	66 (71%)	
Gastric band	1 (0.5%)	14 (14%)	26 (28%)	
Others	2 (1.0%)	2 (2%)	1 (1%)	

TAP, transversus abdominis plane block; s.d., standard deviation; BMI, body mass index; ASA, American Society of Anesthesiologists; COPD, chronic obstructive pulmonary disease. *Continuous variables were analyzed using the independent samples *t*-test; †Categorical variables were analyzed using Fisher’s exact test, where appropriate.

### Primary outcome: LOS

**Fig. 3:**
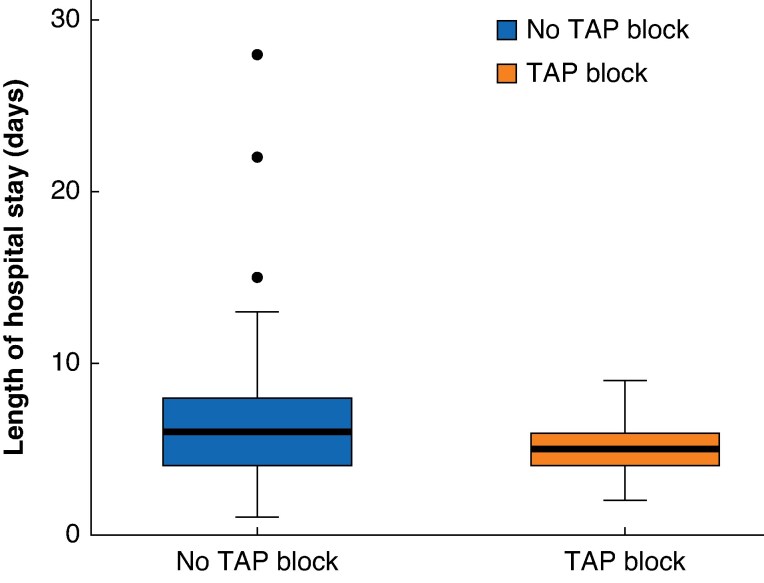
Box plot comparing length hospital of stay between patients who received a TAP block and those who did not Median value (bold centre line), interquartile range (box), and range (error bars) excluding outliers (symbols) are shown. TAP, transversus abdominis plane.

Patients were hospitalized for a mean(s.d.) of 5.86(3.04) days. The average LOS was 4.77 days in the TAP group and 6.89 days in the non-TAP group. Multivariate linear regression revealed a reduction of 2.11 (95% confidence interval (c.i.) −2.92 to −1.35) days in the TAP group compared with the non-TAP group (*P* < 0.001). This effect persisted after adjustment in the *a priori* confounder model (OR −1.88, 95% c.i. −2.73 to −1.02; *P* < 0.001, effect size, Cohen’s *d* = 0.45) (*Fig. 3*).

### Secondary outcomes: surgical complications

Surgical complications occurred in 46.9% of the patients; 62.2% of these were minor complications that did not require further surgical intervention. Among the complications, the most prevalent was wound healing disturbance, which occurred in 39 patients (43.3%). Other notable complications included seromas in 12 patients (13.3%), haematomas in 20 (22.2%), and postoperative bleeding in 9 (10.0%).

Logistic regression analysis revealed that patients who had a TAP block had a significantly lower likelihood of experiencing postoperative complications compared with those who did not (OR 0.48, 95% c.i. 0.27 to 0.86; *P* = 0.012, effect size 0.52). This protective effect persisted after adjusting for confounders in the *a priori* model, showing a 56% reduction in risk (OR 0.43, 0.26 to 0.98; *P* = 0.040).

### Exploratory analyses: most common complications in composite outcome

After primary and secondary results were analysed, it was observed that 15.6% of patients developed postoperative haematoma, 22.2% in the non-TAP group and 8.6% in the TAP group. Logistic regression showed that patients who received a TAP block were 67% less likely to experience this complication (OR 0.33, 95% c.i. 0.14 to 0.78; *P* = 0.012, effect size 0.66). This effect prevailed after adjusting for confounders in the *a priori* confounder model (OR 0.34, 0.13 to 0.89; *P* = 0.031).

Wound healing disturbances were observed in 44.4% of the TAP group and 34.8% of the non-TAP group. Although the TAP group showed fewer instances of postoperative bleeding, the difference was not statistically significant. Similarly, postoperative bleeding occurred in 5.2% of patients, with fewer patients affected in the TAP group, but this difference also did not reach statistical significance.

### Exploratory analysis: reoperation

Revisional surgery was undertaken in 6.8% of all patients. The incidence of acute revisional surgery was higher in the TAP group compared with the non-TAP group (11.1 *versus* 2.2%). Initially, this difference was statistically significant (OR 0.17, 95% c.i. 0.04 to 0.82; *P* = 0.032) but, after adjustment in the *a priori* confounder model, the difference was no longer significant (OR 0.31, 0.07 to 1.49; *P* = 0.151).

### Exploratory analysis: medication use

In the exploratory analysis, the medications administered were investigated to further assess the implications of the aforementioned findings.

After surgery, patients in the TAP group received lower doses of piritramide compared with those in the non-TAP group. Initially, this difference was statistically significant (coefficient −1.70, 95% c.i. −3.39 to −0.001; *P* = 0.030). However, after adjusting for confounders in the *a priori* confounder model, the difference was no longer significant (coefficient −1.60, −3.43 to 0.23; *P* = 0.084).

**Fig. 4:**
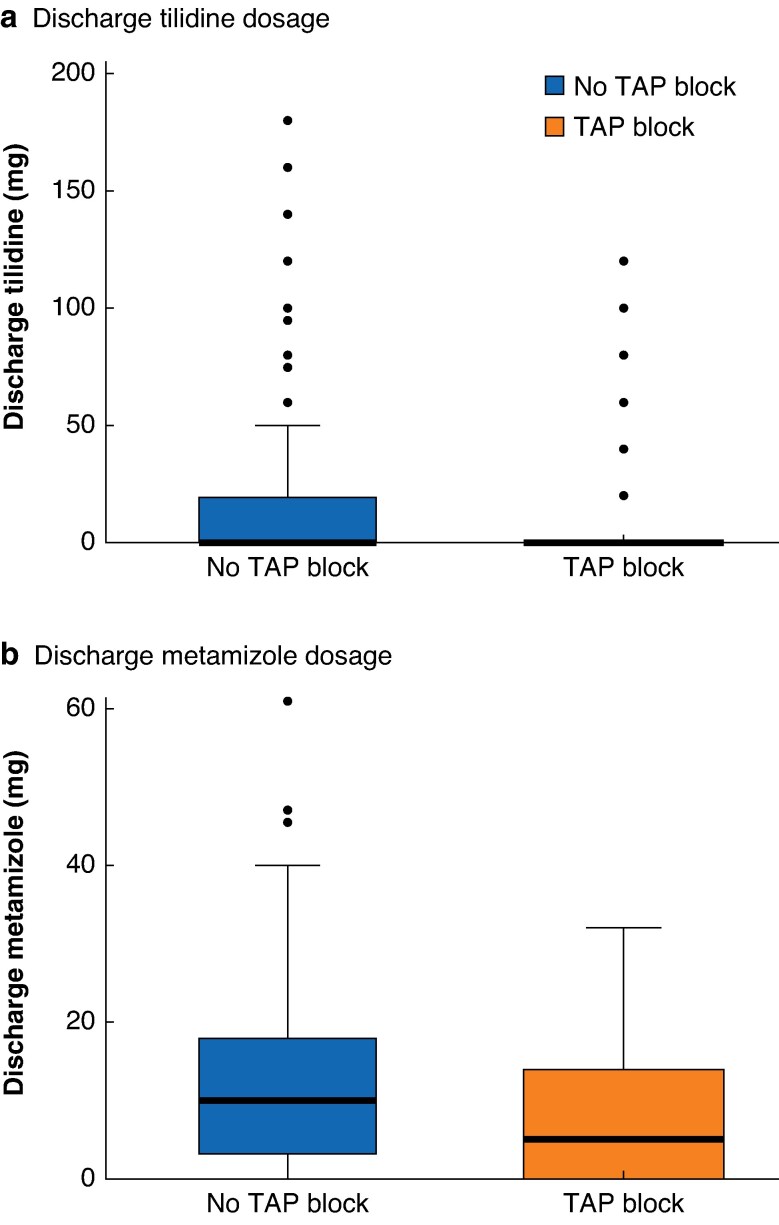
Box plots comparing discharge tilidine and metamizole dosages between patients who received a TAP block and those who did not **a** Discharge tilidine dosage; **b** discharge metamizole dosage. Median value (bold centre line), interquartile range (box), and range (error bars) excluding outliers (symbols) are shown. TAP, transversus abdominis plane.

Patients in the TAP group were prescribed significantly lower doses of tilidine (mean (s.d.) 52.31(32.18) *versus* 59.34(44.39) mg; *P* = 0.024) and metamizole (11.30(7.49) *versus* 15.30(11.93) mg; *P* = 0.032) at discharge. These reductions remained significant after adjustment in the *a priori* confounder model, with a decrease in tilidine prescriptions (coefficient −9.83, −19.17 to −0.49; *P* = 0.038) and a similar reduction in metamizole prescriptions (coefficient −3.61, −6.89 to −0.33; *P* = 0.032). Results are shown in *Fig. 4*.

## Discussion

Benefits of regional nerve blocks after abdominoplasties are well documented in the literature. Nevertheless, there is disagreement regarding the application and the algorithm for the agent to be applied. The present study revealed significant benefits of the regional nerve block extending far past the first postoperative day.

Although the first publication of a TAP block described an ultrasound-guided injection before surgery^24,25^, postoperative or continuous interval applications for 48 h were also common^26^. Some authors^27,28^ introduced the open TAP block technique, performed by plastic surgeons, directly applied during or at the end of the surgery. Others^29,30^ used liposomal bupivacaine as a different carrier. Feng *et al*.^31^ emphasized the efficiency of utilizing a mixture of multiple blocks in the ventral abdominal wall, successfully contributing to pain management.

The main findings of the present analysis demonstrated a clear association between use of the TAP block and reduced LOS in patients who had an abdominoplasty. It enabled earlier mobilization, decreased the risk of overall surgical complications, and decreased the dosage of postoperative pain medications. The finding of regional nerve blocks as adjunct procedures contributing to shorter hospital stay is well established in other surgical procedures^32,33^. There is a paucity of valid data in the literature quantifying the direct cost savings of reducing a single hospital day in the context of abdominoplasties; however, the cost reduction associated with a mean LOS reduction of 2.11 days will be substantial, even without taking into account the cost savings resulting from the prevention of complications related to postoperative haematoma management, such as delayed revisions or increased expenses associated with wound care.

Similar studies^34,35^ have estimated that the cost reduction due to a single day less spent in hospital varies between 200 and 500 euros (€) per day. Notably, the total cost of a TAP block procedure is remarkably low at approximately €50.

The add-on therapy of liposomal bupivacaine (Exparel^®^; Pacira Pharmaceuticals, San Diego, CA, USA) compared with a TAP block alone increases overall cost. However, due to its chemical properties that extend the duration of anaesthetic effects, bupivacaine has the potential to become a valuable adjunct to traditional regional nerve blocks, facilitating the feasibility of performing abdominoplasties in an ambulatory setting in the near future^30,36^.

One of the secondary main findings is the overall reduction in surgical complications in the TAP group, and the decreased appearance of postoperative haematoma could potentially be explained by several factors related to the mechanisms of the TAP block. It provides effective regional anaesthesia, leading to improved pain control and reduced reliance on systemic opioids, which minimizes side-effects like nausea, vomiting, and respiratory depression that can indirectly affect recovery^37–39^. By controlling pain, it stabilizes haemodynamic parameters, reducing the risk of haematoma formation caused by vascular pressure fluctuations. Effective analgesia with reduced tilidine, metamizole, and opioid use, as seen in the present study, also facilitates early postoperative mobilization, improving venous return and reducing complications such as venous stasis. Additionally, Wu *et al*.^40^ demonstrated the anti-inflammatory potential of ropivacaine by indicating significant inhibition of cytokine production (tumour necrosis factor α, interleukin (IL) 6, and IL-1β) in lipopolysaccharide-stimulated RAW 264.7 macrophages and promoting better tissue oxygenation, supporting wound healing, and lowering the risk of complications. Although all TAP blocks were applied under direct vision of the correct plane via ultrasound imaging, it is worth mentioning the toxic potential for chondrotoxicity or systematic effects, for example. Therefore, Jansson^41^ indicated that higher concentrations of ropivacaine can lead to systemic absorption. However, careful use at lower concentrations may optimize vasoconstrictive effects, which help improve the retention of the local anaesthetic and sustain a decreased risk of postoperative haematoma. Chen *et al*.^42^ underlined this statement by advising the combination of ropivacaine and dexamethasone, as it extends the nerve block's duration, with changes in local vasodynamics contributing to this extended vasoconstrictive effect. These factors, combined with potentially fewer intraoperative interventions for haemostasis owing to optimized surgical conditions, might explain the favourable outcomes in the TAP block group.

Further investigation into these mechanisms, potentially through detailed haemodynamic and biochemical analyses, could provide valuable insights into how regional anaesthesia influences tissue healing, pain management, and the prevention of complications such as haematomas and seromas.

The study has several limitations that should be considered. First, as a retrospective cohort study, it is subject to potential biases, such as selection bias and confounding factors. Despite statistical adjustments, the lack of randomization between the TAP and non-TAP groups also means that inherent differences between the groups could have potentially influenced the results. Additionally, unmeasured confounders, like individual variation in surgical technique, variation in practitioner experience, or patient adherence to postoperative care, could have affected the outcomes.

The study’s single-centre design limits the generalizability of the findings, as practices may vary between institutions. Furthermore, in the authors’ institution there was a change in leadership in the anaesthesia and plastic surgery department, which should also be considered as a potential confounder. The decision to use the TAP block was likely influenced by anaesthetist and surgeon preferences, introducing the possibility of selection bias.

Prospective, randomized multicentre studies are essential to explore the mechanisms and long-term benefits of regional nerve blocks in abdominoplasties, including their impact on patient recovery, quality of life, and potential chronic effects of repeated applications. Additionally, comparing different regional anaesthesia techniques, as well as their combined use with adjunct therapies like dexamethasone or liposomal bupivacaine, could further refine treatment protocols and improve surgical outcomes in the long term, potentially confirming the present findings, and addressing these mentioned limitations.

This study has demonstrated that use of the TAP block in abdominoplasty significantly reduces LOS, and decreases the incidence of postoperative complications, as well as the need for postoperative analgesia. These benefits highlight the potential of TAP blocks as a cost-effective, efficient adjunct to abdominoplasty procedures, improving patient outcomes and facilitating faster recovery. Additionally, the reduction in surgical complications, particularly haematomas, may be attributed to the improved pain control and haemodynamic stability provided by the TAP block.

## Data Availability

The data sets generated and/or analysed during the present study are not publicly available but can be obtained from the corresponding author upon reasonable request.
